# Percutaneous Coronary Interventions: Current Innovations and Future Perspectives

**DOI:** 10.3390/jcm15218506

**Published:** 2026-11-02

**Authors:** Quentin Liabot, Aurelia Zimmerli, Benjamin Honton, Adil Salihu, Olivier Muller, Stephane Fournier, David Meier

**Affiliations:** 1 Cardiology Department, Lausanne University Hospital and University of Lausanne, 1005 Lausanne, Switzerland; aurelia.zimmerli@chuv.ch (A.Z.);; 2 Cardiology Department, Clinique Pasteur, 31300 Toulouse, France; bhonton@clinique-pasteur.com

**Keywords:** percutaneous coronary intervention, coronary artery disease, technical innovations, coronary thrombectomy, intracoronary imaging, drug-coated balloons, bioresorbable scaffolds, coronary sinus reducer, artificial intelligence, coronary physiology, coronary microvascular dysfunction

## Abstract

Percutaneous coronary intervention (PCI) has undergone remarkable evolution over the past decades, leading to substantial improvements in procedural success, patient outcomes, and long-term prognosis, although significant challenges remain. Recent technological and therapeutic advances are reshaping contemporary PCI practice. In acute coronary syndromes, novel thrombectomy approaches and emerging reperfusion strategies aim to improve myocardial salvage. In chronic coronary syndromes, the increasing adoption of intracoronary imaging, advanced calcium modification technologies, drug-coated balloons, and next-generation bioresorbable scaffolds is driving the transition toward more individualized and less implant-dependent revascularization strategies. Parallel developments in antithrombotic therapy, including shortened dual antiplatelet therapy (DAPT) regimens, aspirin-free approaches, and personalized treatment algorithms, seek to improve the balance between ischemic protection and bleeding risk. Furthermore, growing recognition of coronary microvascular disease has expanded the focus of PCI beyond epicardial stenoses, highlighting the importance of functional assessment and targeted therapeutic strategies. Finally, artificial intelligence (AI) is emerging as a transformative tool with potential applications in procedural planning, imaging interpretation, physiological assessment, and clinical decision-making. This review summarizes the most relevant contemporary innovations in PCI and discusses future perspectives that are likely to redefine the management of coronary artery disease.

## 1. Introduction

Percutaneous coronary intervention (PCI) has evolved considerably over recent decades, driven by continuous technological and therapeutic advances. Nevertheless, several challenges remain, highlighting the need for more effective and individualized approaches. This review summarizes recent innovations across acute and chronic coronary syndromes and discusses emerging evidence that may shape the future of coronary interventions.

## 2. Current Innovations in Acute Coronary Syndromes

### 2.1. Management of Large Thrombus Burden Beyond Conventional Thrombectomy

Management of thrombus burden has long been a major challenge during primary PCI for ST-segment elevation myocardial infarction (STEMI), as it may compromise both epicardial and microvascular reperfusion. In this setting, thrombus aspiration emerged as an attractive and straightforward adjunctive strategy to reduce thrombus burden before stent implantation.

### 2.2. Failure of Conventional Aspiration Thrombectomy

Early enthusiasm for manual thrombus aspiration was largely driven by the single-center TAPAS trial [1], which demonstrated improved myocardial reperfusion and lower cardiac mortality at one year than conventional PCI (3.6% vs. 6.7%). However, these promising findings were not confirmed by subsequent larger randomized trials. In TASTE [2], involving 7244 STEMI patients, routine manual aspiration failed to reduce 30-day all-cause mortality (2.8% vs. 3.0%; HR 0.94, 95% CI 0.72–1.22; *p* = 0.63), including in the subgroup of patients with high thrombus burden. These neutral findings were further reinforced by TOTAL [3], which included 10,732 patients, and showed identical rates of the primary composite endpoint of cardiovascular death, recurrent myocardial infarction, cardiogenic shock, or NYHA class IV heart failure at 180 days (6.9% vs. 7.0%; HR 0.99, 95% CI 0.85–1.15; *p* = 0.86). Importantly, routine aspiration was associated with a significant increase in stroke at 30 days (0.7% vs. 0.3%; HR 2.06, 95% CI 1.13–3.75; *p* = 0.02). Finally, an individual patient-level meta-analysis pooling these three studies (19,047 randomized patients) confirmed the absence of a significant clinical benefit from routine manual aspiration, although a potential benefit in patients with high thrombus burden could not be excluded [4].

Collectively, these findings shifted the paradigm away from routine manual thrombus aspiration toward selective use in carefully selected cases and prompted the development of more effective thrombus-removal strategies, including powered aspiration systems and mechanical thrombectomy devices, potentially overcoming some of the limitations of conventional manual aspiration.

### 2.3. Emerging Thrombectomy Technologies

The Indigo System CAT RX (Penumbra, Inc., Alameda, CA, USA) is a mechanical aspiration thrombectomy system specifically designed for the removal of thrombus from the coronary arteries. Unlike conventional manual aspiration catheters, in which negative pressure is generated intermittently using a syringe, the CAT RX system provides sustained mechanical aspiration through a dedicated vacuum pump. The system consists of a rapid-exchange aspiration catheter connected to a vacuum pump by aspiration tubing, with an interposed collection canister to collect the aspirated material. The catheter is compatible with a standard 0.014-inch coronary guidewire and a 6Fr guide catheter and incorporates a relatively large aspiration lumen together with a flexible distal segment designed to facilitate navigation through tortuous coronary anatomy. Continuous negative pressure is maintained as the catheter is advanced over the guidewire toward the thrombus, allowing sustained thrombus engagement and extraction rather than the intermittent aspiration achieved with syringe-based systems.

The CAT RX system was clinically evaluated in the prospective, multicenter, single-arm CHEETAH study [5], which enrolled 400 patients with acute coronary syndrome and high thrombus burden (TIMI thrombus grade 4 or 5) undergoing thrombectomy before PCI at 25 US centers. The primary composite safety endpoint occurred in 3.6% of patients, while stroke occurred in 0.77%, with no device-related serious adverse events. At final angiography, TIMI thrombus grade 0, TIMI 3 flow, and myocardial blush grade 3 were achieved in 99.5%, 97.5%, and 99.75% of patients, respectively. These findings established the feasibility and initial clinical safety of sustained mechanical aspiration with CAT RX in selected patients with high thrombus burden, although the single-arm design of CHEETAH precludes conclusions regarding superiority over conventional manual aspiration or PCI without thrombectomy.

Beyond sustained mechanical aspiration, stent-retriever thrombectomy represents another emerging approach to overcome the limitations of conventional thrombus aspiration in patients with large thrombus burden. Originally developed for mechanical thrombectomy in acute ischemic stroke, stent retrievers are self-expanding, retrievable nitinol devices that expand within the thrombus, promote clot integration within the stent structure, and allow subsequent en bloc thrombus extraction. Their application to the coronary circulation was initially described in isolated case reports, predominantly as a bailout strategy for persistent or organized thrombus despite conventional treatment [6,7]. More recent reports have progressively refined the technique by combining mechanical retrieval with aspiration to maximize thrombus extraction while limiting distal or systemic embolization [6,8,9]. In practice, a microcatheter is advanced across the thrombus over a conventional 0.014-inch coronary guidewire. After guidewire removal, the stent retriever is advanced through the microcatheter and deployed across the thrombotic segment by withdrawing the microcatheter. The device is subsequently retrieved into either the guiding catheter or, preferably, a guide-extension catheter positioned immediately proximal to the stent retriever, while continuous aspiration is applied to reduce the risk of embolization [8,9]. Intracoronary imaging may additionally facilitate device sizing and deployment, particularly in large-caliber vessels [6]. Importantly, stent-retriever thrombectomy should not be regarded as a substitute for conventional aspiration in all patients with visible thrombus, but rather as an escalation strategy in selected patients with particularly large, organized or long-standing thrombus, or after anticipated or documented failure of aspiration. A recently proposed stepwise algorithm therefore favors conventional aspiration, with or without glycoprotein IIb/IIIa inhibitors, for fresh, non-organized and low-to-moderate thrombus burden, whereas stent-retriever-based thrombectomy, alone or combined with aspiration, is considered for large, organized or older thrombi and when aspiration is unlikely to succeed or has already failed [9].

Clinical evidence has subsequently expanded beyond individual case reports. In the first prospective coronary experience, Spirito et al. [10] treated 61 patients with acute coronary syndrome and large thrombus burden using the NeVa stent-retriever system, originally developed for neurovascular thrombectomy. The procedure was associated with substantial improvements in epicardial flow, myocardial blush and angiographic thrombus burden, although transient non-flow-limiting and reversible coronary spasm occurred in 23% of patients. The randomized RETRIEVE-AMI trial [11] subsequently compared Solitaire X stent-retriever thrombectomy, manual aspiration and PCI without thrombus modification in 81 patients with STEMI and large angiographic thrombus burden. Although present thrombus volume did not significantly differ between groups, both thrombectomy strategies reduced thrombus burden compared with no thrombus modification, and stent-retriever thrombectomy extracted substantially greater thrombus volumes than manual aspiration, without device-related clinically relevant arterial injury or excess major adverse cardiac or cerebrovascular events at 6 months. Notably, these early studies relied on devices primarily developed for neurovascular interventions, whereas the enVast system (Vesalio) was specifically developed as a coronary stent-retriever platform. This device was evaluated in the multicenter randomized NATURE trial [12], the largest randomized evaluation of coronary stent-retriever thrombectomy to date. Among 156 randomized patients with STEMI, large thrombus burden (TIMI thrombus grade ≥ 3), and presentation within 8 h of symptom onset, enVast thrombectomy followed by conventional PCI significantly reduced the primary endpoint of enzymatic infarct size compared with conventional PCI alone. Exploratory cardiac magnetic resonance (CMR) imaging provided concordant findings, with infarct size representing 17% versus 28% of left ventricular mass, respectively, while no excess in early major adverse cardiovascular events was observed. Although these findings provide encouraging evidence supporting selective stent-retriever thrombectomy in STEMI with large thrombus burden, the available studies remain primarily focused on procedural efficacy. Importantly, improved thrombus extraction or reduced infarct size should not be assumed to translate into better clinical outcomes, and adequately powered randomized trials assessing hard clinical endpoints remain warranted. Beyond coronary thrombectomy, the stent retriever may also offer an off-label bailout option for the retrieval of embolized material, potentially expanding its role in coronary interventions [13]. From a practical standpoint, contemporary coronary thrombectomy devices are generally compatible with conventional 6Fr guiding catheters, allowing their use through standard transradial access. Although no specific excess of access-site complications has been identified, potential device-related risks, including coronary spasm, vessel injury, and distal or systemic embolization, should be considered, particularly given the limited clinical experience with these emerging technologies. The available clinical evidence on coronary stent-retriever thrombectomy is summarized in Table 1.

### 2.4. Myocardial Salvage Beyond Epicardial Reperfusion

Despite successful restoration of epicardial flow, myocardial reperfusion may remain incomplete because of microvascular obstruction (MVO), the pathological substrate of the no-reflow phenomenon. MVO results from the interplay between prolonged ischaemia, spontaneous or procedure-related distal embolisation, reperfusion injury and individual susceptibility, leading to intravascular obstruction through endothelial swelling, platelet–neutrophil plugging and microthrombi, as well as extravascular compression from myocardial oedema, cardiomyocyte hypercontracture, intramyocardial haemorrhage and pericyte constriction [14]. This dissociation between epicardial patency and myocardial reperfusion has prompted the development of adjunctive strategies targeting coronary microcirculation.

Among these, supersaturated oxygen (SSO_2_) therapy delivers hyperoxaemic autologous blood to the infarct territory after primary PCI, aiming to reduce endothelial oedema and improve microvascular perfusion. In AMIHOT II [15], SSO_2_ significantly reduced infarct size in patients with anterior STEMI, although vascular and bleeding complications prompted simplification of the delivery technique. IC-HOT [16,17] subsequently demonstrated the feasibility and favorable safety profile of a 60-min infusion through a 5-Fr catheter positioned in the left main coronary artery. More recently, König et al. [18] provided contemporary mechanistic evidence using multimodality imaging. Among 40 patients with anterior STEMI, SSO_2_ was associated with smaller CMR-defined infarct size (20.4% vs. 32.3%), greater myocardial salvage (47% vs. 19%), less MVO, and higher left ventricular ejection fraction (LVEF) at 6 months. Nevertheless, these encouraging findings remain hypothesis-generating given the small, single-centre, non-randomised and unadjusted design, underscoring the need for adequately powered randomised trials. Supporting this mechanistic signal, a recent meta-analysis of six studies including 1660 patients found that SSO_2_ significantly reduced infarct size and MVO [19]. However, these benefits did not translate into significant reductions in major adverse cardiovascular events (MACE), all-cause mortality, reinfarction, or target-vessel revascularization.

Targeting reperfusion injury before restoration of epicardial flow represents another appealing concept. In EURO-ICE [20], selective intracoronary hypothermia rapidly cooled the jeopardised myocardium before and immediately after reperfusion, but despite being feasible and safe, failed to reduce infarct size compared with primary PCI alone (23.1% vs. 21.6%; *p* = 0.43). Thus, despite encouraging effects on surrogate markers, whether targeting MVO and reperfusion injury can ultimately translate into improved clinical outcomes remains the critical unanswered question.

Taken together, these findings illustrate both the biological potential and the persistent challenges of translating strategies targeting MVO and reperfusion injury into clinically meaningful myocardial salvage after primary PCI. Importantly, whether the favorable effects of SSO_2_ on infarct size and MVO translate into improved clinical outcomes is currently being investigated in HOT-AAMI [21], a multicentre randomised trial enrolling 1266 patients with anterior STEMI and powered for a primary composite endpoint of all-cause mortality and heart failure events.

## 3. Current Innovations in Chronic Coronary Syndromes

### 3.1. Intracoronary Imaging-Guided PCI

Over the past decade, intracoronary imaging has evolved from a primarily diagnostic adjunct to an integral component of PCI optimization, particularly for complex coronary lesions. Unlike angiography, which provides only a two-dimensional luminogram, intravascular ultrasound (IVUS) and optical coherence tomography (OCT) enable cross-sectional assessment of vessel dimensions, plaque morphology, lesion preparation, stent expansion and apposition, and procedure-related complications. Although intracoronary imaging is also valuable in acute coronary syndromes [22,23], its use is particularly established in chronic coronary syndromes, where it is most commonly applied in clinical practice and supported by the largest body of evidence.

IVUS relies on ultrasound, providing relatively deep tissue penetration and allowing real-time imaging without contrast injection, albeit with a lower spatial resolution (approximately 100 μm). Conversely, OCT uses near-infrared light and provides approximately ten-fold higher resolution (10 μm), enabling detailed characterization of plaque and stent architecture, at the expense of shallower tissue penetration and the need for transient blood clearance, usually with contrast injection. Both IVUS and OCT can be performed through conventional 6Fr guiding catheters using transradial access. Their use does not appear to confer a substantial additional risk of access-site complications, although potential device-related coronary complications and the additional contrast requirements of OCT should be considered.

Clinical evidence initially accumulated predominantly for IVUS. IVUS-XPL [24] demonstrated that IVUS guidance reduced MACE after PCI of long lesions, an advantage maintained at 5 years [25], while ULTIMATE [26] subsequently extended this benefit to an all-comer population, with lower target-vessel failure (TVF) at both 1 and 3 years [27]. In parallel, RENOVATE-COMPLEX-PCI [28], in which IVUS accounted for nearly three quarters of imaging-guided procedures, demonstrated a significant reduction in MACE across a broad spectrum of complex lesions. In contrast, the recent European IVUS-CHIP trial [29] failed to demonstrate superiority of systematic IVUS guidance in complex high-risk PCI, with non-significant higher TVF in the IVUS group (13.9% vs. 11.1%; HR 1.25; *p* = 0.08). Importantly, pre-PCI IVUS was performed in only 64% of lesions, predefined optimization criteria were achieved in just 48%, and outcomes in the angiography arm were better than anticipated, potentially limiting the ability to demonstrate incremental benefit. These neutral findings highlight the importance of effective imaging-guided optimization and should be interpreted in the context of the broader randomized evidence supporting intracoronary imaging in complex PCI.

In parallel, evidence supporting OCT has expanded rapidly. ILUMIEN IV [30] demonstrated greater final minimal stent area with OCT guidance, although this procedural improvement did not translate into significantly lower TVF at 2 years. In contrast, OCTOBER [31] demonstrated a significant reduction in MACE after OCT-guided PCI of complex bifurcations, while OCCUPI [32] subsequently extended this clinical benefit to a broader population with complex lesions. Beyond routine stent optimization, OCT may be particularly valuable in specific anatomical settings. In CALIPSO [33], an OCT-based algorithm for calcified lesions resulted in substantially greater stent expansion than angiographic guidance, whereas the RESTO registry [34] highlighted its ability to identify the mechanisms of in-stent restenosis, including underexpansion, neoatherosclerosis, and neointimal hyperplasia, modifying the treatment strategy in approximately one third of patients.

Collectively, these data have moved intracoronary imaging into contemporary clinical practice, with the 2024 ESC guidelines giving IVUS or OCT a Class I, Level A recommendation for PCI of anatomically complex lesions, including left main, true bifurcations, and long lesions [35]. More recently, an updated meta-analysis including > 20,000 patients [36] further confirmed the clinical benefit of intracoronary imaging-guided PCI, reinforcing its prominent role in contemporary guidelines.

Future developments may further reshape this paradigm. Ultra-high-resolution photon-counting coronary CT could increasingly permit detailed non-invasive characterization of calcified disease, stents, and coronary anatomy before PCI, while integration of intracoronary imaging with physiological information may ultimately provide a combined anatomical and functional framework for procedural planning and optimization. Major randomized trials evaluating intracoronary imaging-guided PCI are summarized in Table 2 and Table 3.

### 3.2. Calcium Modification Technologies

Moderate-to-severe coronary calcification is encountered in approximately 20% of patients undergoing PCI and remains a major determinant of procedural complexity, impairing device delivery and stent expansion and increasing the risk of subsequent stent failure. Beyond calcium detection, intracoronary imaging provides detailed assessment of its distribution and morphology, thereby allowing lesion preparation to be tailored to the underlying calcium phenotype and linking imaging guidance directly to calcium-modification strategies [37].

**Rotational atherectomy** (RA) uses a high-speed diamond-coated burr to preferentially ablate rigid calcified tissue through differential cutting and remains particularly useful for severely calcified, balloon-uncrossable lesions, especially long and proximal lesions with predominantly superficial or protruding calcium, including nodules. Although PREPARE-CALC [38] demonstrated greater procedural success with RA than modified balloons, randomized evidence supporting systematic atherectomy remains limited.

**Orbital atherectomy** (OA) similarly modifies superficial calcium using an eccentrically mounted diamond-coated crown whose orbital diameter increases with rotational speed [39]. Its bidirectional operation and orbital mechanism may offer advantages in long, proximal or tortuous lesions, with potential effects extending beyond superficial calcium. However, the large ECLIPSE trial [40] found no significant improvement in either minimal stent area or 1-year TVF with routine OA compared with a balloon-based strategy, supporting selective rather than systematic use when lesions are balloon-crossable and dilatable.

**Excimer laser coronary angioplasty** (ELCA) combines photochemical, photothermal and photomechanical effects and, unlike dedicated atherectomy systems, can be delivered over a conventional 0.014-inch guidewire. Its direct effect on calcium is limited, making ELCA particularly attractive for balloon-uncrossable fibrocalcific lesions, resistant in-stent restenosis and selected underexpanded stents rather than extensive deep calcification [39]. In the randomized ROLLER COASTR-EPIC22 trial [41], IVL was non-inferior to RA for stent expansion, whereas ELCA did not meet the prespecified non-inferiority criterion.

**Intravascular lithotripsy** (IVL) has substantially expanded the therapeutic armamentarium. Conventional Shockwave IVL generates acoustic pressure waves that propagate circumferentially and transmurally, fracturing both superficial and deep calcium with minimal dependence on wire bias [37]. It is particularly suited to balloon-crossable lesions with extensive concentric or deep calcification, although the limited balloon length (12 mm) makes it more convenient for focal lesions. The pooled DISRUPT CAD studies [42] reported 92.4% procedural success with low angiographic complication rates, while the recent randomized ICARE-OFDI trial [43] demonstrated non-inferiority of IVL versus RA for final minimal stent area, with similar 12-month target-lesion failure (TLF). More recently, the LithiX Hertz Contact IVL system introduced mechanical lithotripsy without an external energy source, using metallic hemispheres mounted on a semi-compliant balloon to concentrate mechanical stress onto calcium. In the first-in-human PINNACLE-I study [44], calcium fractures were observed in 90.6% of lesions, with 96.7% mean stent expansion at the minimum stent area and only 1.7% MACE at 30 days, providing an innovative mechanical approach to calcium modification that warrants evaluation in larger randomized trials. Nevertheless, the randomized Short-Cut (NCT06089135) and VICTORY (NCT05346068) trials demonstrated the non-inferiority of well-established technologies, namely cutting balloon angioplasty and super-high-pressure non-compliant balloon angioplasty, compared with IVL for lesion preparation and stent expansion in calcified coronary lesions.

**Figure 1:**
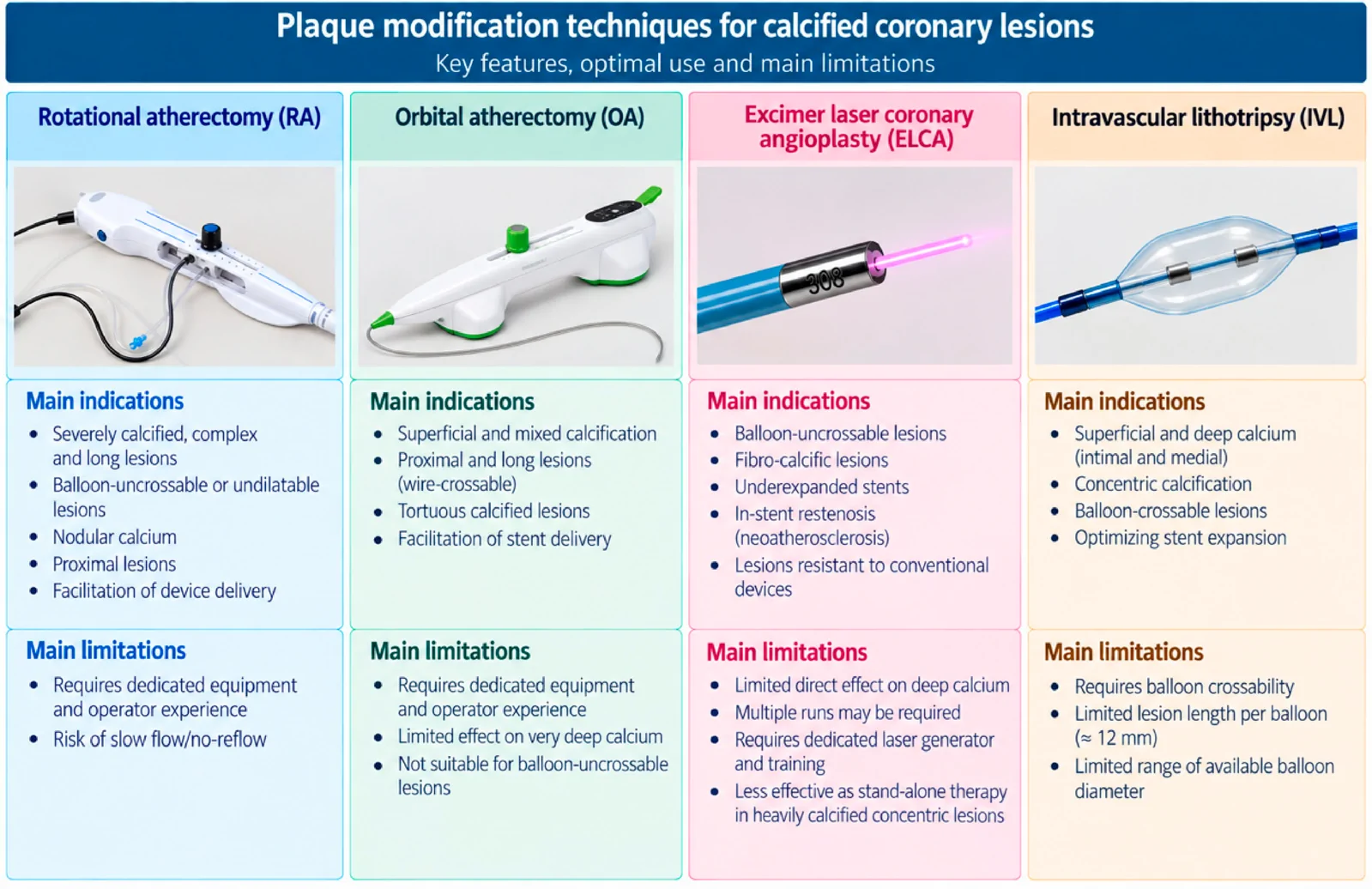
Plaque modification techniques for calcified coronary lesions. Key features, optimal use and main limitations.

Consequently, the strength of evidence supporting different calcium-modification technologies remains heterogeneous, favoring a tailored approach based on patient and calcium characteristics rather than their systematic use. Moreover, the selection of calcium-modification technologies should also account for their specific procedural risks. Rotational and orbital atherectomy may be associated with coronary dissection, perforation, and slow-flow/no-reflow, whereas ELCA also carries a risk of dissection and perforation, with complication risk varying according to the underlying lesion characteristics [45]. IVL generally exhibits a favorable safety profile, with low rates of angiographic complications, although dissection and perforation remain possible. All of these techniques can be performed through conventional 6Fr transradial access, although guiding catheter size and vascular access should be tailored to lesion complexity. Accordingly, careful lesion assessment, preferably using intracoronary imaging, remains essential to optimize device selection and minimize procedural complications. The main indications and limitations of currently available calcium-modification techniques are summarized in Figure 1.

### 3.3. The Stentless Revolution: Back to the Future?

#### 3.3.1. Drug-Coated Balloons

Drug-coated balloons (DCB) have revived the concept of stentless PCI by combining mechanical dilatation with local delivery of an antiproliferative drug, without leaving a permanent metallic scaffold. Beyond avoiding additional metal layers, this “leave-nothing-behind” strategy may preserve coronary vasomotion, eliminate the risk of late stent-related events, and potentially allow shorter antiplatelet regimens. However, the absence of mechanical scaffolding makes outcomes highly dependent on adequate lesion preparation and exposes patients to acute recoil or flow-limiting dissection requiring bailout stenting.

In-stent restenosis (ISR) remains the most established indication, particularly when avoiding additional metallic layers is desirable. The DAEDALUS individual-patient meta-analysis [46] showed comparable outcomes between DCB and drug-eluting stents (DES) for bare-metal stent ISR, whereas DES reduced TLR in DES-ISR, highlighting a trade-off between avoiding further stent layers and greater antirestenotic efficacy. More recently, SELUTION4ISR [47] demonstrated non-inferiority of a sirolimus-coated balloon compared with a mixed standard-of-care strategy, although outcomes remained less favorable than DES in single-layer ISR.

Small-vessel disease represents another particularly attractive indication, as permanent scaffolding further reduces an already limited lumen. In BASKET-SMALL 2 [48], DCB was non-inferior to second-generation DES, with comparable outcomes maintained at 3 years [49], while the ANDROMEDA individual-patient data meta-analysis [50] further supported the long-term safety and efficacy of DCB in vessels ≤ 3 mm. The absence of a permanent implant and the possibility of shorter antiplatelet therapy may also make DCB particularly attractive in patients at high bleeding risk, with a prespecified BASKET-SMALL 2 analysis [51] showing comparable efficacy to DES irrespective of bleeding risk.

Conversely, extending DCB-only PCI to larger de novo lesions remains more controversial. REC-CAGEFREE I [52] failed to demonstrate non-inferiority versus systematic DES implantation, mainly because of more frequent repeat revascularization, whereas the larger SELUTION DeNovo trial [53] subsequently demonstrated non-inferiority of a sirolimus-coated balloon strategy at 1 year, albeit with approximately one-fifth of patients requiring stenting. Variations in patient selection, lesion preparation, and bailout stenting strategies may also contribute to the conflicting results of REC-CAGEFREE I and SELUTION DeNovo. Notably, the particularly favorable outcomes in the DES control arm of REC-CAGEFREE I may have made non-inferiority more difficult to demonstrate, whereas approximately 20% of patients in SELUTION DeNovo required stent implantation. These findings further emphasize that DCB-only strategies should be evaluated according to the specific device, lesion characteristics, and procedural strategy rather than extrapolated across different platforms.

Consequently, DCB should not be regarded as a homogeneous device class. Unlike conventional paclitaxel-coated balloons, which rely on rapid tissue uptake and prolonged intracellular retention of a highly lipophilic drug, the SELUTION SLR sirolimus-coated balloon incorporates sirolimus into biodegradable micro-reservoirs transferred to the vessel wall through a phospholipid-based cell-adherent technology, enabling sustained drug release. This technological shift is particularly relevant for sirolimus, whose lower lipophilicity and reversible tissue binding make effective balloon-based delivery more challenging than with paclitaxel. Accordingly, clinical performance may depend not only on the antiproliferative agent itself, but also on coating formulation, drug-transfer efficiency, and tissue-retention kinetics, arguing against a uniform class effect across DCB platforms [54].

Furthermore, DCB may be particularly appealing for side-branch treatment during provisional bifurcation PCI [55] and as part of hybrid strategies in chronic total occlusion (CTO) PCI, where reducing the length and number of implanted stents may be advantageous, although evidence in CTO remains predominantly registry-based [56,57]. The recently launched BASKET BALL trial (NCT07363161) will further challenge the boundaries of the “leave-nothing-behind” concept by comparing a sirolimus-coated balloon-only strategy with upfront DES implantation in patients at increased clinical or anatomical risk, integrating both target-vessel failure and major bleeding into its clinical assessment.

Finally, in selected acute coronary syndromes, the REVELATION trial [58,59] provided proof-of-concept for a stentless approach, although thrombus burden, plaque instability, recoil, and dissection remain important concerns.

#### 3.3.2. Bioresorbable Scaffolds

Bioresorbable scaffolds (BRS) were developed to reconcile the acute mechanical benefits of stenting with the long-term advantages of a “leave-nothing-behind” strategy. By providing transient radial support and drug delivery during vascular healing before undergoing complete resorption, BRS were expected to avoid the long-term consequences of permanent metallic caging, including chronic inflammation, neoatherosclerosis and late stent-related events, while potentially restoring physiological vasomotion, adaptive remodeling, and future options for percutaneous or surgical revascularization [60]. However, the initial enthusiasm surrounding first-generation polymeric scaffolds was tempered by the failure of the Absorb bioresorbable vascular scaffold, which was associated with higher rates of TLF and scaffold thrombosis than contemporary DES [60,61]. These adverse outcomes resulted from a combination of device- and procedure-related limitations, including thick polymeric struts, impaired deliverability, disturbed coronary flow and delayed endothelialization, insufficient radial strength, non-uniform degradation and scaffold dismantling, as well as suboptimal vessel sizing, expansion and implantation technique [60].

Rather than invalidating the concept itself, these limitations have driven the development of a new generation of BRS with improved mechanical properties, thinner struts and more predictable resorption profiles. Particular interest has shifted toward resorbable magnesium scaffolds, which combine the mechanical properties of a metallic platform with subsequent bioresorption. The third-generation sirolimus-eluting Freesolve scaffold incorporates an optimized magnesium alloy with enhanced radial strength. In the first-in-human BIOMAG-I study [62,63], 1-year TLF was 2.6%, with no cardiac death or definite/probable scaffold thrombosis. Its clinical performance is now being tested against a contemporary everolimus-eluting DES in BIOMAG-II (NCT05540223), a randomized non-inferiority trial that has completed enrollment of 1861 patients, with target-lesion failure at 12 months as the primary endpoint. Nevertheless, despite encouraging early results, robust long-term comparative data remain limited, and whether newer magnesium-based scaffolds can overcome the late safety concerns observed with first-generation polymeric platforms remains to be established through adequately powered randomized trials and extended follow-up.

Beyond replacing metallic DES on a one-to-one basis, BRS may ultimately enable entirely new revascularization strategies. The ongoing investigator-initiated BIOHYBRID trial (NCT06710210) explores this concept in long and diffuse coronary disease by combining a Freesolve magnesium scaffold in larger proximal segments requiring mechanical support with paclitaxel-coated balloons in the remaining smaller distal diseased segments, thereby minimizing permanent coronary implants.

#### 3.3.3. Coronary Sinus Reducer

The coronary sinus reducer (CSR) represents a conceptually distinct approach to the treatment of refractory angina, targeting the coronary venous rather than the arterial circulation. It is primarily intended for patients with persistent angina and documented myocardial ischemia despite optimal medical therapy who are not amenable to further surgical or percutaneous revascularization. The hourglass-shaped device is implanted percutaneously within the coronary sinus, creating a controlled focal narrowing that increases coronary venous pressure [64]. Although its precise mechanism of action remains incompletely understood, increased venous pressure is thought to promote capillary recruitment, reduce microvascular resistance, and redistribute blood flow from the relatively well-perfused subepicardium toward the more vulnerable ischemic subendocardium.

Clinical evidence was initially established by the randomized, double-blind, sham-controlled COSIRA trial [65], in which 104 patients with refractory CCS class III–IV angina were randomized to CSR implantation or a sham procedure. At 6 months, improvement by ≥2 CCS classes occurred in 35% versus 15% of patients, respectively, with a significant improvement in quality of life. These findings were subsequently supported by the large, prospective REDUCER-I real-world registry [66,67], which confirmed high procedural success and sustained improvements in angina severity and quality of life. More recently, the randomized sham-controlled ORBITA-COSMIC trial [68] provided important mechanistic insights: despite a significant reduction in daily angina episodes, CSR implantation did not significantly increase myocardial blood flow in ischemic segments compared with placebo, suggesting that its symptomatic benefit may involve more complex changes in myocardial perfusion distribution or microvascular physiology rather than an increase in global myocardial blood flow. Ongoing randomized trials, including COSIRA-II (NCT05102019), REMEDY (NCT05492110) and COSIMA (NCT04606459), are expected to further define the clinical efficacy, mechanisms of action, and optimal patient selection for CSR therapy, while also exploring its potential role beyond obstructive coronary artery disease, particularly in patients with coronary microvascular dysfunction.

## 4. Antithrombotic Therapy: Less Is More?

The contemporary management of antiplatelet therapy after PCI is progressively shifting from standardized treatment durations towards dynamic, patient-tailored strategies balancing ischaemic and bleeding risks over time. This concept is supported by the distinct temporal patterns of these competing hazards: thrombotic risk is highest immediately after PCI and declines thereafter, whereas bleeding risk persists throughout treatment, providing a rationale for potent antiplatelet protection during the early phase followed by subsequent treatment simplification [69,70]. Accordingly, shortening DAPT and early aspirin withdrawal followed by P2Y12 inhibitor monotherapy have emerged as attractive strategies. In TWILIGHT [71], discontinuation of aspirin after 3 months of uneventful DAPT reduced BARC 2, 3, or 5 bleeding by 44% without increasing death, myocardial infarction, or stroke, while TICO [72] extended this concept to acute coronary syndrome (ACS) patients, demonstrating fewer net adverse clinical events with ticagrelor monotherapy after 3 months of DAPT. Importantly, while TICO exclusively enrolled ACS patients (one-third with STEMI and two-thirds with non-ST-elevation ACS), TWILIGHT included approximately two-thirds of patients with non-ST-elevation ACS (STEMI were excluded) and one-third with stable coronary artery disease. These differences are clinically relevant, as the underlying presentation may influence the optimal timing and selection of antiplatelet strategies, reflecting differences in baseline thrombotic risk.

More recently, TARGET-FIRST [73] pushed this strategy further: among 1942 selected patients with ACS, complete revascularization, and an event-free first month, P2Y12 inhibitor monotherapy after only 1 month of DAPT was non-inferior to continued DAPT for the primary composite endpoint (2.1% vs. 2.2%) while markedly reducing clinically relevant bleeding (2.6% vs. 5.6%; HR 0.46, 95% CI 0.29–0.75). However, how early aspirin can safely be withdrawn remains critical. In NEO-MINDSET [74], discontinuation of aspirin within 4 days after PCI in 3410 ACS patients failed to demonstrate non-inferiority for ischaemic events despite substantially reducing bleeding, with subsequent analyses showing that the excess ischaemic risk was concentrated within the first 30 days. Importantly, 62% of patients presented with STEMI, and a prespecified subgroup analysis suggested that the excess ischemic risk associated with early aspirin withdrawal was predominantly observed in STEMI rather than non-ST-elevation ACS [75]. These complementary findings suggest that the first month after ACS PCI remains a particularly vulnerable thrombotic period and support 1-month DAPT as a potential threshold before aspirin withdrawal in appropriately selected patients rather than systematic immediate aspirin-free therapy.

An alternative approach is P2Y12 inhibitor de-escalation, preserving potent platelet inhibition during this early high-risk period while subsequently reducing bleeding liability. In TOPIC [76], switching from ticagrelor or prasugrel to clopidogrel 1 month after ACS reduced net adverse clinical events (13.4% vs. 26.3%), predominantly through fewer bleeding events, without a significant increase in ischaemic events.

Ultimately, these strategies should not be viewed as competing fixed regimens but as components of a personalized antiplatelet continuum, integrating clinical presentation, procedural complexity, bleeding and ischaemic risks, and individual treatment response to provide greater antithrombotic intensity when thrombotic protection is most needed and progressively simplify therapy as bleeding risk becomes predominant. In this evolving landscape, the growing use of DCB and the potential resurgence of BRS may further reshape antithrombotic management toward more patient-tailored strategies.

## 5. Artificial Intelligence and Coronary Physiology

The assessment of myocardial ischemia is increasingly supported by computational technologies that integrate anatomical and physiological information. Artificial intelligence (AI) and computational physiological modelling are evolving approaches that can complement one another in the analysis of coronary anatomy and function. While AI enables automated image analysis and data-driven predictions, computational modelling allows the estimation of coronary haemodynamics through mathematical simulations of blood flow. Together, these approaches are expanding the possibilities for deriving physiological information from coronary imaging and supporting the assessment of the functional significance of coronary artery disease.

Coronary physiology, including the invasive measurement of fractional flow reserve (FFR) and resting physiological indices, has significantly improved the assessment of the hemodynamic significance of coronary stenoses. However, invasive physiological assessment requires the use of pressure wires and, in the case of FFR, pharmacological hyperemia. These requirements increase procedural complexity, duration, and cost and may limit the widespread adoption of invasive physiological assessment [77,78].

### 5.1. Angiography-Derived FFR

Virtual assessment of fractional flow reserve (FFR) from coronary angiography enables the haemodynamic significance of intermediate coronary lesions to be assessed without the need for a pressure wire or pharmacological hyperaemia.

Several angiography-derived physiological methods are currently available, using different computational approaches and image acquisition requirements, including quantitative flow ratio (QFR), FFRangio, and vessel fractional flow reserve (vFFR; CAAS vFFR). The fundamental principle underlying these angiography-derived FFR techniques is the three-dimensional reconstruction of the coronary anatomy from at least two angiographic projections acquired at different viewing angles, typically separated by 25–30°, to minimise vessel foreshortening and overlap.

### 5.2. Quantitative Flow Ratio (QFR)

Following three-dimensional reconstruction of the coronary artery, the pressure drop across the vessel is estimated using computational fluid dynamics (CFD), with coronary flow velocity derived from the TIMI frame count in contrast-QFR. The diagnostic accuracy of QFR compared with invasive FFR was demonstrated in the FAVOR III China trial, in which QFR achieved a diagnostic accuracy of 86% for the detection of FFR ≤ 0.80 [79]. Regarding clinical outcomes, the FAVOR III Europe trial randomised 2000 patients to a QFR-guided or an invasive FFR-guided strategy. The trial did not meet the prespecified criteria for non-inferiority with respect to the composite endpoint of death, myocardial infarction, or unplanned revascularisation at 12 months [80]. At 2 years, the rate of major adverse cardiac events remained numerically higher in the QFR-guided group than in the FFR-guided group (9.7% vs. 7.4%; HR 1.34, 95% CI 0.98–1.81; *p* = 0.064). The excess risk was concentrated in the first year, with similar event rates between groups from 1 to 2 years [81].

### 5.3. FFRangio

FFRangio uses a fixed-flow CFD model, with real-time aortic pressure obtained from the guiding catheter used as an input parameter. This approach generates a full-coronary-tree FFR map. In the FAST-FFR study, FFRangio demonstrated a diagnostic accuracy of 92% compared with invasive pressure-wire-derived FFR [82]. More recently, the ALL-RISE trial demonstrated that an FFRangio-guided strategy was non-inferior to an invasive pressure-wire-guided strategy with respect to the composite endpoint of death, myocardial infarction, or unplanned revascularisation at 1 year [83].

### 5.4. Vessel Fractional Flow Reserve (vFFR)

vFFR is based on three-dimensional quantitative coronary angiography (3D-QCA) and uses a fixed-flow computational fluid dynamics (CFD) model, with invasively measured aortic root pressure used as the inlet boundary condition. In the FAST II study, vFFR demonstrated excellent diagnostic performance compared with invasive FFR, with an area under the curve of 0.93 and a diagnostic accuracy of 90% [84]. More recently, the FAST III randomised trial demonstrated that a vFFR-guided revascularisation strategy was non-inferior to an FFR-guided strategy with respect to the composite endpoint of death, myocardial infarction, or any revascularisation at 1 year [85].

By deriving FFR directly from routinely acquired coronary angiographic images, these approaches have the potential to facilitate the broader adoption of physiological lesion assessment in everyday clinical practice. The main characteristics of angiography-derived FFR techniques are summarized in Table 4.

### 5.5. CCTA-Derived FFR

Fractional flow reserve derived from CCTA (FFR-CT) enables non-invasive assessment of the hemodynamic significance of coronary stenoses by combining anatomical information from CCTA with computational modeling of coronary blood flow.

**Figure 2:**
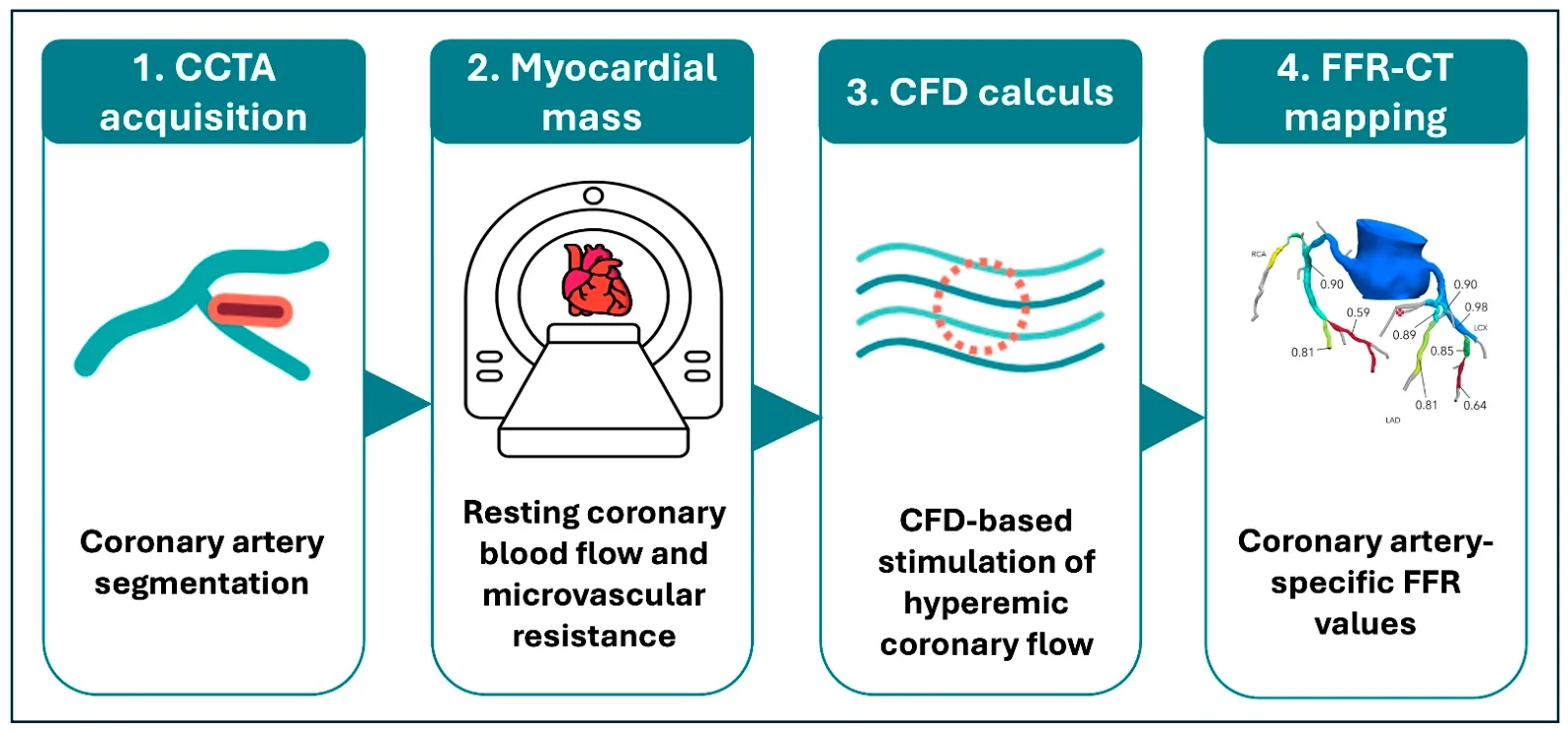
FFR-CT computational workflow from CCTA acquisition to CFD-based simulation and coronary artery-specific FFR-CT mapping. CCTA: coronary computed tomography angiography; CFD: computational fluid dynamics; FFR-CT: computed tomography-derived fractional flow reserve.

FFR-CT computation involves four main steps: (1) coronary artery segmentation from high-resolution CCTA images; (2) estimation of resting coronary blood flow and microvascular resistance based on CCTA-derived myocardial mass and coronary vessel dimensions; (3) computational fluid dynamics (CFD)-based simulation of maximal hyperemia and coronary blood flow using the Navier–Stokes equations; and (4) generation of coronary artery-specific FFR-CT maps [86] (Figure 2).

The clinical validation of FFR-CT has been extensively investigated in patients with stable coronary artery disease. The DISCOVER-FLOW trial was among the first studies to demonstrate the diagnostic performance of FFR-CT against invasive FFR, supporting the feasibility of non-invasive physiological assessment derived from coronary anatomical imaging [87]. More recently, the IMPACT FFR study showed that incorporating FFR-CT into the diagnostic pathway was associated with fewer invasive diagnostic procedures and revascularizations, without an observed difference in 1-year clinical outcomes [88].

While the role of FFR-CT in stable coronary artery disease has been extensively investigated, its application in acute coronary syndromes remains less well established. A recent study evaluated the potential role of FFR-CT to evaluate the culprit lesion in high-risk patients with non-ST-segment elevation acute coronary syndrome (NSTE-ACS) [89]. FFR-CT showed improved diagnostic performance compared with CCTA alone, particularly at the lesion level, suggesting that physiological information derived from CCTA may provide additional diagnostic value in this high-risk population [90].

The potential role of FFR-CT in acute coronary syndromes may extend beyond the assessment of the suspected culprit lesion. A sub-study analysis investigated whether FFR-CT could identify non-culprit lesions with hemodynamic significance and therefore potentially help determine which lesions may require revascularization [91].

By integrating anatomical and physiological information within a single non-invasive assessment, FFR-CT may help refine patient selection for invasive coronary angiography and reduce unnecessary invasive procedures. Further validation in acute coronary syndromes and in more complex clinical settings will be essential to determine its role in the next generation of coronary disease management.

## 6. Coronary Microcirculation: The Next Frontier

Despite major advances in the diagnosis of epicardial coronary artery disease, a substantial proportion of patients undergoing coronary angiography for angina have no obstructive coronary stenosis. For many years, symptoms in these patients were frequently considered to be of non-cardiac origin. However, the absence of obstructive epicardial coronary artery disease does not exclude coronary vascular dysfunction. Indeed, in approximately half of patients with angina and non-obstructive coronary arteries, symptoms may be attributed to distinct vasomotor endotypes, including coronary vasospasm and coronary microvascular dysfunction (CMD) [92,93]. Accurate identification of these conditions is clinically important because they are associated with impaired quality of life, adverse cardiovascular outcomes, and substantial healthcare utilization [94,95,96].

### 6.1. Coronary Vasospasm

#### 6.1.1. Pathophysiology

Coronary vasospasm results from an inappropriate increase in coronary vascular smooth-muscle constriction and may involve either the epicardial coronary arteries or the coronary microcirculation [97]. Although epicardial coronary vasospasm has been recognized for decades, microvascular spasm remains less well characterized and is increasingly recognized as an important mechanism of angina in patients with non-obstructive coronary arteries.

#### 6.1.2. Diagnosis

Invasive acetylcholine (ACh) provocation testing is the reference method for assessing coronary vasomotor disorders and distinguishing epicardial from microvascular spasm. The COVADIS criteria provide standardized definitions for these entities [98]. Epicardial coronary spasm is characterized by transient ≥ 90% luminal constriction in association with reproduction of the patient’s typical angina and ischemic electrocardiographic changes. In contrast, microvascular spasm is characterized by reproduction of typical anginal symptoms and ischemic electrocardiographic changes in the absence of significant epicardial coronary constriction.

Several studies have investigated clinical and angiographic characteristics associated with a positive ACh provocation test, including female sex, endothelial dysfunction, the presence of epicardial atherosclerosis, and coronary tortuosity [99,100,101]. However, these findings remain inconsistent and have rarely been externally validated. A recent retrospective study evaluating clinical predictors of ACh-induced coronary spasm found that age ≥ 60 years and hyperlipidemia were associated with a lower prevalence of inducible spasm [102]. Further studies are therefore required to improve the identification of patients who are most likely to benefit from invasive coronary vasomotor testing.

#### 6.1.3. Treatment

The treatment of coronary vasospasm is primarily based on preventing inappropriate coronary vasoconstriction. Calcium-channel blockers (CCBs) represent the first-line therapy, and high doses may be required in patients with severe or refractory vasospastic angina. In selected patients, a combination of a dihydropyridine and a non-dihydropyridine CCB may be considered [35].

Pharmacological strategies targeting alternative vasoconstrictive pathways are also being investigated. The PRIZE trial evaluated the endothelin-A receptor antagonist zibotentan in patients with microvascular angina [103]. However, zibotentan did not improve exercise duration or secondary clinical outcomes and was associated with a higher incidence of adverse events, highlighting the difficulty of translating promising pathophysiological targets into effective therapies for coronary microvascular disease [104].

### 6.2. Coronary Microvascular Dysfunction (CMD)

#### 6.2.1. Pathophysiology

**Figure 3:**
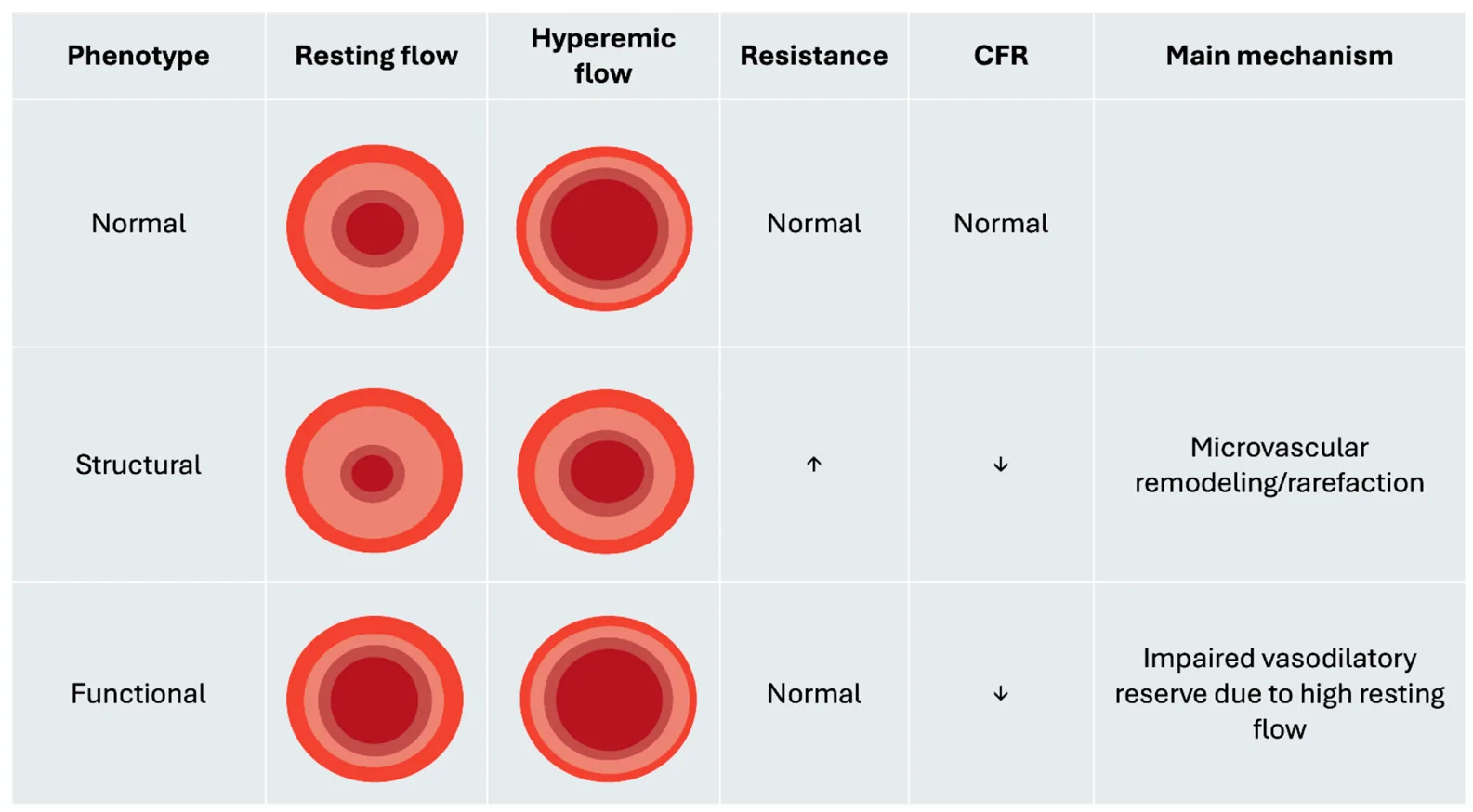
Hemodynamic profiles of coronary microvascular dysfunction phenotypes. Schematic representation of normal, structural, and functional phenotypes according to resting and hyperemic coronary blood flow, microvascular resistance, and coronary flow reserve (CFR). Circle size represents relative coronary blood flow.

CMD encompasses several pathophysiological phenotypes that may involve either impaired microvascular vasodilatory capacity or increased microvascular resistance. A useful conceptual distinction can be made between functional and structural CMD (Figure 3).

Functional CMD may be characterized by an increased resting coronary flow, resulting in a reduced CFR despite preserved microvascular resistance [105].

In contrast, structural CMD is characterized by an increase in microvascular resistance, reflecting structural abnormalities of the coronary microcirculation, including arteriolar remodeling and capillary rarefaction. These alterations limit coronary blood flow during hyperemia and result in both increased microvascular resistance and reduced CFR [106].

Coronary blood flow and microvascular resistance are strongly influenced by the amount of myocardium supplied by the interrogated coronary artery [107]. Consequently, differences in the subtended myocardial mass may influence the interpretation of coronary flow and resistance measurements. A recent study investigated the relationship between subtended myocardial mass, coronary flow, and microvascular resistance using coronary computed tomography angiography (CCTA) to quantify the myocardial territory supplied by the interrogated vessel [108]. Despite a significantly smaller subtended myocardial mass, patients with structural CMD exhibited higher microvascular resistance and lower hyperemic coronary flow than control subjects, even after accounting for the myocardial territory supplied. In contrast, patients with functional CMD had a myocardial mass comparable to that of control subjects and did not demonstrate a significant limitation of hyperemic coronary flow. These findings provide important pathophysiological insights into the different CMD phenotypes and challenge the interpretation of reduced CFR as a uniform marker of CMD. They support the concept that impaired hyperemic flow and increased microvascular resistance are key mechanisms underlying symptoms in structural CMD, whereas the mechanisms responsible for symptoms in functional CMD remain less clearly defined.

#### 6.2.2. Diagnosis

The diagnosis and characterization of CMD require assessment of coronary vasodilatory capacity and microvascular resistance. Coronary flow reserve (CFR) provides an integrated measure of the ability of the coronary circulation to increase blood flow in response to hyperemia and is calculated as the ratio of hyperemic to resting coronary blood flow.

Coronary microvascular function can be assessed non-invasively using transthoracic Doppler echocardiography, cardiac magnetic resonance imaging (CMR), or positron emission tomography (PET). However, invasive coronary function testing provides a comprehensive approach to characterizing coronary microvascular and vasomotor abnormalities during coronary angiography.

For many years, invasive assessment of coronary microvascular function was primarily based on bolus thermodilution, allowing calculation of CFR and the index of microvascular resistance (IMR). More recently, continuous intracoronary thermodilution has enabled the quantitative assessment of absolute coronary blood flow and microvascular resistance [109,110,111]. This approach is based on continuous infusion of saline into the coronary artery and measurement of changes in blood temperature, allowing estimation of absolute coronary blood flow. When combined with distal coronary pressure measurements obtained using a pressure wire, absolute microvascular resistance can also be calculated.

The 2024 European Society of Cardiology (ESC) Guidelines consider a CFR < 2.5 to be abnormal in patients with non-obstructive coronary arteries, although the appropriate threshold may vary according to the measurement technique and clinical context [35].

Regarding microvascular resistance, an IMR ≥ 25 is generally considered indicative of increased microvascular resistance. The microvascular resistance reserve (MRR), which integrates changes in microvascular resistance between resting and hyperemic conditions, provides an additional physiological parameter and may complement CFR and IMR in the characterization of CMD. Absolute hyperemic microvascular resistance measured by continuous thermodilution has also been investigated, with values around 475–500 WU proposed as potential thresholds for abnormal microvascular resistance. However, unlike IMR, these thresholds remain less extensively validated and should therefore be interpreted with caution [106,112].

#### 6.2.3. Treatment

The treatment of CMD remains challenging because available evidence is based largely on relatively small and heterogeneous studies, and the number of adequately powered randomized trials evaluating specific pharmacological strategies remains limited.

Importantly, the CorMicA trial demonstrated that invasive coronary function testing followed by stratified medical therapy according to the underlying endotype can improve angina symptoms and quality of life [113]. These findings support an endotype-based therapeutic approach rather than the use of a uniform treatment strategy for all patients with angina and non-obstructive coronary arteries.

The effects of individual pharmacological therapies on coronary microvascular function remain less well established. For example, a recent study evaluating CCB monotherapy did not demonstrate a significant improvement in invasive measures of coronary microvascular function [114].

Current European recommendations advocate an individualized therapeutic strategy according to the underlying coronary endotype and cardiovascular risk profile. In patients with CMD and impaired vasodilatory capacity or increased microvascular resistance, treatment options include beta-blockers, CCBs, angiotensin-converting enzyme inhibitors (ACE inhibitors), and ranolazine, with statins used according to the patient’s overall cardiovascular risk and the presence of atherosclerotic disease [35]. The EAPCI Expert Consensus Document similarly proposes a stepwise approach combining risk-factor modification with antianginal therapy, including beta-blockers, ACE inhibitors, CCBs, and ranolazine, according to the clinical phenotype and response to treatment [115].

Nevertheless, the optimal pharmacological treatment of CMD remains uncertain. Future randomized trials should determine whether endotype-guided and mechanism-targeted treatment strategies can improve not only symptoms but also objective measures of coronary microvascular function. In this context, the STAR-INOCA study is evaluating whether state-of-the-art medical therapy based on the EAPCI consensus recommendations can improve invasive measures of coronary microvascular function [116].

## 7. Conclusions

PCI is moving toward more precise, individualized, and less implant-dependent care. Recent innovations are broadening therapeutic possibilities and challenging established paradigms, although many still require confirmation. Their successful translation into clinical practice will depend on careful patient selection and a balanced assessment of benefits and risks. Continued scientific and technological progress is likely to further reshape coronary intervention in the coming years.

## Figures and Tables

**Table 1:** Current clinical evidence on coronary stent-retriever thrombectomy.

First Author	Year	Study Design	Number of Patients	Device	Concomitant Aspiration	Strategy	Main Findings
D’Entremont [6]	2024	Case report	1	Solitaire X	Yes—continuous CAT RX aspiration	Bailout after failed aspiration	Complete thrombus extraction and TIMI 3 flow
Walsh [7]	2025	Case report	1	Solitaire X	No	Adjunctive after balloon-mediated flow restoration	Complete thrombus clearance
Liabot [8]	2026	Case report	1	enVast	Yes—continuous CAT RX aspiration	Bailout after failed aspiration	Complete thrombus removal despite prior aspiration failure
Chong [9]	2026	Case report	1	enVast	Yes—continuous CAT RX aspiration	First-line hybrid thrombectomy	Complete thrombus removal
Spirito [10]	2022	Prospective, single-arm study	61	NeVa	Vacuum-assisted aspiration for 51 patients	First-line	Feasible and effective; improved TIMI flow, myocardial blush and thrombus grade
Kotronias [11]	2025	RCT	81	Solitaire X	Yes—syringe aspiration	First-line	Feasible and safe; greater thrombus extraction than manual aspiration
Valgimigli [12]	2026	RCT	156	enVast	No	First-line	Reduced infarct size vs. PCI alone; no early safety signal

PCI: percutaneous coronary intervention; RCT: randomized clinical trial; TIMI: thrombolysis in myocardial infarction.

**Table 2:** Major randomized trials evaluating IVUS-guided PCI.

Trial	First Author (Year)	Number of Patients	Lesion Subset	Comparison	Primary Endpoint	Main Findings/ Conclusions
IVUS-XPL [24]	Hong (2015)	1400	Long lesions (stent length > 28 mm)	IVUS vs. angiography	MACE at 1 year: cardiac death, target-lesion MI, or ischemia-driven TLR	2.9% vs. 5.8%; HR 0.48; *p* = 0.007 IVUS improved 1-year outcomes after long lesions PCI
ULTIMATE [26]	Zhang (2018)	1448	All-comer DES PCI	IVUS vs. angiography	TVF at 12 months: cardiac death, target-vessel MI, or clinically driven TVR	2.9% vs. 5.4%; HR 0.53; *p* = 0.019. IVUS-guided DES implantation significantly improved clinical outcome in all-comers
RENOVATE-COMPLEX-PCI [28]	Lee (2023)	1639	Complex PCI: long lesions, CTO, LM, calcified, ostial lesions, ISR	Intracoronary imaging (73% IVUS) vs. angiography	TVF at 2 years: cardiac death, target-vessel MI, or clinically driven TVR	7.7% vs. 12.3%; HR 0.64; *p* = 0.008. Imaging-guidance improved clinical outcomes in complex PCI
IVUS-CHIP [29]	Diletti (2026)	2020	Complex high-risk PCI: long lesions, bifurcations, LM, CTO, calcified, ostial lesions, ISR	IVUS vs. angiography	TVF at 19 months: cardiac death, target-vessel MI, or clinically driven TVR	13.9% vs. 11.1%; HR 1.25; *p* = 0.08. Routine IVUS-guided PCI did not reduce TVF

CTO: chronic total occlusion; DES: drug-eluting stent; ISR: in-stent restenosis; IVUS: intravascular ultrasound; LM: left main; MACE: major adverse cardiovascular event; PCI: percutaneous coronary intervention; TVF: target-vessel failure; TLR/TVR: target lesion/vessel revascularisation.

**Table 3:** Major randomized trials evaluating OCT-guided PCI.

Trial	First Author (Year)	Number of Patients	Lesion Subset	Comparison	Primary Endpoint	Main Findings/ Conclusions
ILUMIEN IV [30]	Ali (2023)	2487	Complex coronary lesions	OCT vs. angiography	Post-PCI MSA and TVF at 2 years	5.72 vs. 5.36 mm^2^; *p* < 0.001 MSA was significantly greater with OCT No significant reduction in TVF Less stent thrombosis with OCT
OCTOBER [31]	Holm (2023)	1201	Complex coronary bifurcation (including LM)	OCT vs. angiography	MACE at 2 years: cardiac death, target-lesion MI, or ischemia-driven TLR	10.1% vs. 14.1%; HR 0.70; *p* = 0.035 OCT guidance improved clinical outcomes after complex bifurcation PCI
OCCUPI [32]	Hong (2024)	1604	Complex lesions: long, calcified, bifurcations, LM, CTO	OCT vs. angiography	MACE at 1 year	5% vs. 7%; HR 0.62; *p* = 0.023 OCT guidance reduced 1-year MACE
CALIPSO [33]	Amabile (2025)	143 randomized patients (134 analysed patients)	Moderate-to-severe calcified lesions	OCT-guided algorithm vs. angiography	Post-PCI MSA	6.5 vs. 5 mm^2^; *p* < 0.001 OCT-guided PCI improved stent expansion

CTO: chronic total occlusion; LM: left main; MACE: major adverse cardiovascular events; MI: myocardial infarction; MSA: minimal stent area; OCT: optical coherence tomography; PCI: percutaneous coronary intervention; TLR: target-lesion revascularisation; TVF: target-vessel failure.

**Table 4:** Comparison of QFR, FFRangio and vFFR.

Characteristic	QFR	FFRangio	vFFR
Underlying principle	Three-dimensional coronary artery reconstruction and estimation of pressure drop using CFD.	Fixed-flow CFD model generating a full-coronary-tree FFR map.	Three-dimensional quantitative coronary angiography (3D-QCA) combined with a fixed-flow CFD model.
Coronary flow estimation	Flow velocity estimated from the TIMI frame count in contrast-QFR.	Fixed-flow model.	Fixed-flow model.
Main diagnostic validation study	FAVOR III China	FAST-FFR	FAST II
Diagnostic accuracy vs. invasive FFR	86%	92%	90%
Main randomized clinical trial	FAVOR III Europe	ALL-RISE	FAST III
Clinical comparator	Invasive FFR-guided strategy	Pressure-wire-derived FFR-guided strategy	Invasive FFR-guided strategy
Clinical outcome at 1 year	Non-inferiority was not demonstrated according to the prespecified criteria.	Non-inferiority was demonstrated.	Non-inferiority was demonstrated.
Composite clinical endpoint	Death, myocardial infarction, or unplanned revascularisation.	Death, myocardial infarction, or unplanned revascularisation.	Death, myocardial infarction, or any revascularisation.

QFR: quantitative flow reserve; FFR: fractional flow reserve; vFFR: vessel fractional flow reserve; CFD: computational fluid dynamics.

## Data Availability

No new data were created or analyzed in this study. Data sharing is not applicable to this article.
